# The Effect of the Intensity of Happy Expression on Social Perception of Chinese Faces

**DOI:** 10.3389/fpsyg.2021.638398

**Published:** 2021-06-14

**Authors:** Yaning Li, Zhongqing Jiang, Yisheng Yang, Haizhou Leng, Fuhua Pei, Qi Wu

**Affiliations:** ^1^School of Psychology, Inner Mongolia Normal University, Hohhot, China; ^2^College of Psychology, Liaoning Normal University, Dalian, China; ^3^Department of Elementary Education, Hebei Normal University, Shijiazhuang, China

**Keywords:** happy expression, social perception, intensity, Chinese faces, trait impression

## Abstract

Numerous studies have shown that facial expressions influence trait impressions in the Western context. There are cultural differences in the perception and recognition rules of different intensities of happy expressions, and researchers have only explored the influence of the intensity of happy expressions on a few facial traits (warmth, trustworthiness, and competence). Therefore, we examined the effect of different intensities of Chinese happy expressions on the social perception of faces from 11 traits, namely trustworthiness, responsibility, attractiveness, sociability, confidence, intelligence, aggressiveness, dominance, competence, warmth, and tenacity. In this study, participants were asked to view a series of photographs of faces with high-intensity or low-intensity happy expressions and rate the 11 traits on a 7-point Likert scale (1 = “*not very ××*,” 7 = “*very ××*”). The results indicated that high-intensity happy expression had higher-rated scores for sociability and warmth but lower scores for dominance, aggressiveness, intelligence, and competence than the low-intensity happy expression; there was no significant difference in the rated scores for trustworthiness, attractiveness, responsibility, confidence, and tenacity between the high-intensity and low-intensity happy expressions. These results suggested that, compared to the low-intensity happy expression, the high-intensity happy expression will enhance the perceptual outcome of the traits related to approachability, reduce the perceptual outcome of traits related to capability, and have no significant effect on trustworthiness, attractiveness, responsibility, confidence, and tenacity.

## Introduction

Cultural wisdom warns us not to judge a book by its cover. This suggests that the natural inclination is to judge people by their appearance. Indeed, when meeting strangers for the first time, people infer many characteristics about them based on their facial information (e.g., facial expressions), even in 34 ms (Willis and Todorov, 2006; Todorov et al., 2015). This inference process is called “social perception of faces” (Oosterhof and Todorov, 2008), and the inference results can affect the decisions of people, such as mate selection (Olivola et al., 2014; Valentine et al., 2014), trial outcomes (Wilson and Rule, 2015; Jaeger et al., 2020), and election outcomes (Na and Huh, 2016; Wong and Zeng, 2017).

### Cultural Similarity and Difference in the Social Perception of Faces

Recently, researchers have started to model the structure underlying the social perception of faces. Oosterhof and Todorov (2008) used the trait assessment task to identify two evaluative dimensions: (1) valence related to approach-avoidance and (2) dominance related to physical strength-weakness. Based on the principal component analysis, the trustworthiness score can be used as the representative of the valence dimension, which refers to the behavioral intention of the target face to benefit or harm others. On the other hand, the dominance dimension refers to the ability of the target face to harm others (Oosterhof and Todorov, 2008). Wu et al. (2020) recruited local Chinese participants and used the trait assessment task to identify an approach-avoidance dimension, which was held cross-culturally, as well as a broader “capability” dimension that included dominance and tenacity related to physical and intellectual strength. The rating of the “capability” dimension was crucial for the survival of individuals and to obtain resources and a high social status, which might be considered more typical in collective societies such as China.

Additionally, the top-down stereotype content model has established that perceived warmth and competence are the two universal dimensions of human social cognition both at the individual and group levels. The warmth dimension includes traits that relate to perceived intent, which aligns with the approach-avoidance dimension which includes trustworthiness (Fiske et al., 2007). However, some researchers proposed the “morality differentiation hypothesis,” which suggests that trustworthiness and warmth are separate dimensions. These researchers define trustworthiness related to morality as the behavioral intention to categorize others as either enemies or friends. Conversely, warmth, considered unrelated to morality, has been defined as the proficiency of an individual in recruiting support for their intentions (Goodwin et al., 2014; Landy et al., 2016; Oliveira et al., 2020). Although others do not strongly argue for such distinction and consider trustworthiness and sociability as subcomponents of the warmth dimension, according to them, trustworthiness related to morality can be viewed to be distinct from, and primary compared to, sociability. Sociability implies being benevolent to people in ways that facilitate affectionate relations with them, but trustworthiness refers to being benevolent to people in ways that facilitate correct and principled relations with them (Brambilla et al., 2011, 2012; Brambilla and Leach, 2014). However, because the stereotype content model (two-dimension theories) agglomerated moral and amoral traits within a single dimension, people do not predict that the moral relevance of traits (as opposed to their warmth relevance) should have any special importance for person perception, and the omission of this information from two-dimensional models may therefore lead to a loss of predictive power (Goodwin et al., 2014).

Similar to the warmth-competence stereotype content models, the approach-avoidance dimension in the social perception of faces also agglomerated moral and amoral traits (e.g., trustworthiness and sociability) within a single dimension (Oosterhof and Todorov, 2008; Todorov et al., 2015; Todorov and Oh, 2021), and this might also obscure the information from the moral-relevance and warmth-relevance of traits, which would not reflect their special importance for person perception. Therefore, in the present study, we used the traits assessment task to rate multiple traits rather than dimensions and to explore the effects of happy expression intensity on the social perception of Chinese faces, which would provide more information about the social perception of Chinese faces. This would be a novel perspective in the study of the first impression of strange faces in the Chinese context to explore the “morality differentiation hypothesis.”

### The Effect of Happy Expressions on the Social Perception of Faces

Facial cues in the social perception of faces include immutable (e.g., identity, gender, and race) and variable (e.g., expressions) cues (Haxby et al., 2000). In contrast to immutable cues, variable facial expressions provide critical clues while the social perception of faces is formed (Sutherland et al., 2017). In daily life, happy and neutral expressions are most frequently present on the faces of people. Compared to neutral expressions, happy expressions increase face value in interpersonal communication, resulting in a halo effect. This is the tendency for the positive traits of an individual to “overflow” into additional trait areas in perceptions of others of them (Thompson and Meltzer, 1964). Smiling faces have been rated as more trustworthy, attractive, and popular (Hehman et al., 2019; Li et al., 2020), and less aggressive (Oosterhof and Todorov, 2008). Previous research indicates that facial expressions influence the perception of a single specific dimension of trustworthiness (Caulfield et al., 2016; Sandy et al., 2017), dominance (Kim et al., 2016; Ueda and Yoshikawa, 2018), warmth (Wang et al., 2017), and capability (Beall, 2007; Gao et al., 2016). However, a few studies have directly evaluated the expression effects of multiple traits. Referring to the research by Li et al. (2020) and Wu et al. (2020) was the first group to directly compare the effects of happy Chinese expressions on multiple traits. The results indicated that the evaluation scores of trustworthiness and warmth regarding happy facial expressions varied, which supported the “morality differentiation hypothesis.” These results indicate that it is necessary to explore the effects of happy expressions on multiple traits rather than just single dimensions of the social perception of faces.

In the context of Western culture, mounting evidence indicates that happy expressions of different intensities convey different types of social information. Researchers believe that the intensity of expression corresponds to the intensity of behavioral tendencies (Ekman et al., 1980). Studies have reported that, compared with neutral facial expressions, happy facial expressions at different intensities (25 and 50%) increase the perception scores of trustworthiness among children above 10 years old and that the degree of influence proportionally increases with emotional intensity (Hess et al., 2000; Caulfield et al., 2014, 2016). Furthermore, compared to a low-intensity smile, a high-intensity teeth-showing smile increases the friendly and approachable behavioral tendency of the face, enhancing affinity to the individual. When people are eager to build cooperative relationships with others (Mehu et al., 2008; Bell et al., 2017) or are in search of harmonious interpersonal relationships (Hennig-Thurau et al., 2006), they tend to display a wider smile. Rhesus monkeys also display a toothy smile in subordinate environments, a defensive gesture showing friendly intentions (de Waal and Luttrell, 1985); whereas the bared-teeth display of chimpanzees communicates a benign and non-aggressive intent in affiliate environments (Parr and Waller, 2006). Thus, positive traits associated with sociality (e.g., trustworthiness, submissiveness, and warmth) have been positively correlated with the intensity of happy expressions. However, grins are considered to signal incapability. For example, professional fighters who laugh in pre-match photos are perceived to be less aggressive, less dominant, and more likely to lose than low-intensity smiling fighters (Kraus and Chen, 2013).

While many studies have been conducted on the influence of the intensity of happy expressions on the social perception of faces in Western culture, there are numerous necessary reasons for studying how expression intensity influences Eastern cultures. First, it should be noted that cultural differences exist in the frequency and rules of happy expressions. For example, when comparing photos of Western and Eastern leaders before and after elections, it was found that regardless of the election results, Western leaders presented a high-intensity smile, while Eastern leaders presented a calm and weak smile (Tsai et al., 2016; Fang et al., 2019). Furthermore, when articulating happy expressions *via* texting, Westerners often use parenthesis and a colon, such as in :-) or :), to exaggerate the mouth and reduce the eyes, respectively. In contrast, Easterners often use emoticons, such as (^.^) or (^_^), where the mouth is simplified but the eyes are expressive (Liu et al., 2010). Second, it should also be noted that cultural differences exist in the interpretation of happy expressions. For example, Chinese people believe that a smiling face signals emotional instability, while Americans do not (Walker et al., 2011). Third, although the “approachability” dimension displays cross-cultural consistency (Sutherland et al., 2018; Wu et al., 2020; Jones et al., 2021), contradictory perspectives exist regarding how the meaning of trustworthiness and warmth in the “approachability” dimension is interpreted. In the context of Western culture, some researchers believed that the meanings of these two traits are similar (Fiske et al., 2007; Wang et al., 2019), while others supported the “morality differentiation hypothesis” (Goodwin et al., 2014; Landy et al., 2016). Trustworthiness focuses on morality, while warmth focuses on social interaction (Wang and Cui, 2003), which has been supported by comparing the scores of the traits in happy and neutral expressions (Li et al., 2020). This suggests that displaying happy expressions might be a possible way to separate the two traits, but previous research still lacks relevant in-depth exploration. Additionally, content differences exist in the “capability” dimension of the social perception of the Chinese faces model and the “dominance” dimension of the valence-dominance model. Fourth, in previous studies, researchers used composite software that combined images of neutral and happy facial expressions in different proportions to form experimental materials with two different physical strengths (25 and 50%; Caulfield et al., 2014, 2016). For example, 25% of happy expressions were a 75/25 combination of neutral and happy expressions. The researchers then used the materials to investigate how the intensity of happy expressions affected the social perception of faces. However, the physical intensity of happy expressions did not strictly correspond to its perceived emotional intensity (Hess et al., 2000; Caulfield et al., 2016). Moreover, the composite images were more likely different from the natural faces that participants would encounter in daily life; therefore, they might not have matched with the mental representations of the participants (Hu et al., 2018). It is thus necessary to compare the influence of different intensities of happy expressions on the social perception of faces in the Chinese context with more natural photos.

### The Present Study

In the present study, we investigated the effect of different intensities of happy expressions on the social perception of Chinese faces, which has not been previously addressed. We selected a series of high- and low-intensity happy face images. Participants were asked to rate these face images according to the traits of trustworthiness, responsibility, attractiveness, sociability, confidence, intelligence, aggressiveness, dominance, competence, warmth, and tenacity. These traits were derived from the study by Wu et al. (2020), and we chose 11 of them instead of 15 for the following reasons: these 11 traits had high internal consistency and overlapped with the traits included in the studies of Fiske et al. (2007) and Oosterhof and Todorov (2008), so they could be used as representative traits in the study of social perception of faces. The four traits of masculinity, femininity, emotional stability, and likeability were not included. The traits of masculinity and femininity were excluded because Wu et al. (2020) performed the principal component analysis of traits without the femininity and masculinity ratings. The trait of emotional stability was excluded due to low internal consistency (Li et al., 2020). Likeability was excluded to avoid overlap with sociability (Li et al., 2020; Brambilla et al., 2021). Although the cultural consensus regarding the meaning of the “approachability” dimension, based on the “moral differentiation hypothesis,” we hypothesized that the intensity of happy expressions had different effects on trustworthiness-related traits and warmth-related traits (Hypothesis 1). In addition, since Chinese leaders presented calm, weak smiles in political elections, we hypothesized that low-intensity happy expressions would be rated as more capable than high-intensity happy expressions (Hypothesis 2).

## Materials and Methods

### Participants

A total of 32 Chinese college students aged 18–25 years (16 males and 16 females, mean age 22.06 ± 2.17 years) from Liaoning Normal University participated in the face photo trait-rating experiment. All participants reported normal or corrected-to-normal visual acuity and normal color vision, claimed to be free of current and previous neurological and psychiatric disorders and were not currently using psychotropic medication. All participants were right-handed according to a self-report questionnaire. The sample size for the main study (*N* = 27) was considered appropriate to conduct a 2 × 2 repeated-measures ANOVA since the focus was on the main effect of only one variable (Brysbaert, 2019). The present study only focused on the main effect of the happy expression intensity; therefore, *post hoc* analysis was performed using the G∗Power software. The analysis indicated that the sample of the study (*N* = 32) was sufficient to detect an effect size of *f* = 0.40 (median effect) with a power of 1 − β = 0.8 (Brysbaert, 2019). All participants provided written informed consent and were paid CHN¥40 for their participation in the 1 h experiment. The study was previously approved by the Academic Ethics Committee of Liaoning Normal University.

### Stimuli

#### Stimuli Development

A total of 76 smiling face photos were randomly selected from the Taiwan Facial Expression Image Database (TFEID; Chen and Yen, 2007). These photos were recorded from 38 Chinese people (19 males and 19 females). Two photographs were taken of each individual, one depicting a high-intensity smile and the other a low-intensity smile.

**Figure 1:**
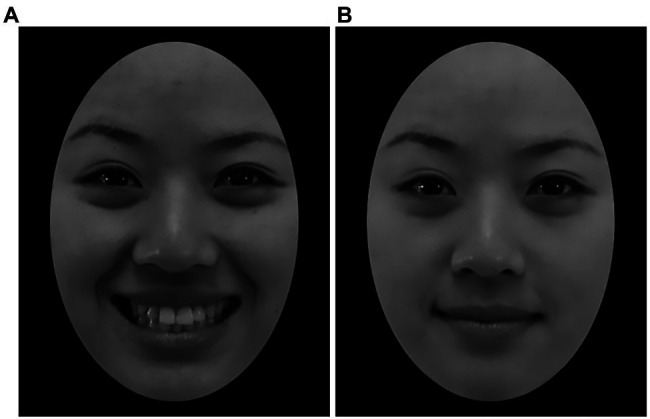
Example of the experimental picture. **(A)** High-intensity happy expression. **(B)** Low-intensity happy expression.

To have enough stimuli for the formal experiment, another 56 smiling face images were collected by taking photos of 28 additional Chinese college students (14 males and 14 females, mean age = 24.46 ± 1.45 years) using the procedure defined by Chen and Yen (2007) for TFEID. Before their photos were taken, the participants were shown sample photos of happy expressions in different intensities (high-intensity and low-intensity; selected from the TFEID, facial recognition rate >90%). The sample photos of happy expressions were formed according to the instructions of the Facial Action Coding System (FACS; Ekman et al., 2002). The facial movements of happy expression included (a) pushed-up cheeks, in which skin gathers under the eye, and a narrowed eye aperture and (b) pulled-up lip corners. Prior literature has determined that at a muscular level, smile intensity is indicated by the amplitude of the zygomatic major movement (the muscle group responsible for pulling the lips upwards; Ekman, 1993). Happy expressions of different intensities are mainly different in their zygomatic major movement levels. A low-intensity happy expression displays a slight contraction of the zygomatic major, which is not enough to show the teeth; a high-intensity happy expression involves displaying an intense contraction of the zygomatic major, which leads to a toothy smile. Participants relaxed their facial muscles and made corresponding expressions by imitating facial muscle movements of happy expressions of different intensities, as depicted in the photos. The location and light were identical, the participants wore the same clothes (white lab coat), the camera parameters were fixed (ISO 1600, 1/100 s, F/4.5), the camera was parallel to the faces of the participants, and the distance from the camera to the participant was 150 cm. After taking the photos, the image standardization process was also conducted according to the criteria of TFEID using Adobe Photoshop (Adobe, 2018) to remove the hair, ears, neck, accessories, and other external features, leaving only facial information. The unified image size was 480 pixels × 600 pixels, and a 4.05 cm × 5.85 cm black circle was applied around each face so that each face only displayed the internal information of the face, such as the eyebrows, eyes, nose, and mouth (example shown in Figure 1).

#### Stimuli Validation

To ensure that the photos presented happy expressions with a high- or low-intensity smile, a screening assessment was conducted. An additional 30 college students were recruited (15 males and 15 females, mean age = 22.17 ± 2.45 years) to assess all 132 photos that were selected from the TFEID and taken by the lab of the researchers. All participants were unfamiliar with the faces in the photos.

The program for assessment was compiled and presented in E-prime 2.0 (PST, 2013), and then divided into two phases: a practice phase (eight trials) and a formal phase (132 trials). The procedure in both phases was identical. For the practice phase, eight additional photos of Chinese people were selected from the TFEID, but they were not used in the formal phase. All eight selected photos corresponded to an emotion type including anger, sadness, fear, happiness, disgust, surprise, contempt, and a neutral expression. For the formal phase, the stimuli were selected from the TFEID and taken from the lab. The participants were tasked with judging the expression type and rating the intensity level of the face. After participants reached a 90% accuracy rate of judging the facial expression type in the practice phase, they entered the formal phase. If the participants failed to reach the 90% threshold within the practice phase, then they remained in that phase. The average number of practice trials was eight.

In each trial, the fixation point was initially presented for 1,000 ms, and facial expression photos were then presented randomly. For each face photo, the participants were first requested to classify the emotion types of the faces by pressing one of eight emotions labeled on the keys of a numeric keypad (1 for “angry,” 2 for “sad,” 3 for “fear,” 4 for “happy,” 5 for “disgusting,” 6 for “surprise,” 7 for “contempt,” and 8 for “neutral”). They were then asked to rate the emotional intensity of the faces on a 9-point Likert scale ranging from 0 (*no emotion*) to 8 (*very strong emotion*) on the alphanumeric keys. The image disappeared after the participant pressed the button, which was followed by a blank screen for 1,000 ms. The participants were given a 30-s break after completing 50 trials before they continued the experiment.

After collecting the assessment data, the recognition accuracy and intensity of happy expressions of each participant were calculated. Recognition accuracy denotes the percentage of the number of photos rated as the happy expression type compared to the total number of photos. Happy emotional intensity refers to the average value of the emotional intensity scores of all photos. A normal distribution test and homogeneity of variance test were conducted for the recognition accuracy and emotional intensity of TFEID, as well as for the newly collected images. The results indicated that the data satisfied normal distribution (Kolmogorov–Smirnov: *p* > 0.05) and homogeneity of variance (Levene’s statistic: *p* > 0.05). SPSS 24.0 software (IBM, 2018) was used to perform paired *t*-tests on the means of the recognition accuracy and intensity of happy expressions for the image sources and facial gender obtained from the responses of the same participants (*N* = 30). The results displayed no significant differences in the image sources (i.e., the TFEID facial expressions and photographed facial expressions by the lab of the researchers; as shown in Table 1) or facial gender (as shown in Table 2); this indicates that the images taken by the lab of the authors were equivalent to the TFEID images.

**Table 1:** Evaluation scores of stimuli from different sources (M ± SD).

	Taiwan emotional faces	Emotional faces photographed	*df*	*t*	*p*	Cohen’s *d*
Recognition accuracy	0.94 ± 0.09	0.93 ± 0.10	29	1.96	0.060	0.36
Intensity	3.96 ± 0.89	3.88 ± 0.91	29	1.95	0.061	0.36

*p < 0.05 was considered statistically significant*.

**Table 2:** Evaluation scores of stimuli from different facial gender (M ± SD).

	Male faces	Female faces	*df*	*t*	*p*	Cohen’s *d*
Recognition accuracy	0.93 ± 0.10	0.95 ± 0.09	29	−1.69	0.101	0.31
Intensity	3.94 ± 0.87	3.90 ± 0.92	29	0.99	0.332	0.18

*p < 0.05 was considered statistically significant*.

Additionally, the 132 face photos of 66 people that were taken (one photo with a high-intensity smile and another with a low-intensity smile for each person) were divided into two equal groups. In each group, no persons were represented in more than one face photo, and thus, the participants did not view two photos of the same person. These two groups of face photos were used separately to compile one version of a trait rating program; this was done to avoid interference with the identity information in the trait rating. Thus, Version 1 of the trait rating program included the face photos of 19 people with high-intensity happy expressions from TFEID and the face photos of 14 people with high-intensity happy expressions from the photos taken by the lab. The photos with low-intensity happy expressions of these 33 people were assigned to Version 2. The other photos were assigned in this same way among Versions 1 and 2.

After dividing the photos into two versions, paired *t*-tests were conducted on the means of recognition accuracy and intensity of happy expressions for the two versions. The results showed significant differences for the high- and low-intensity smiling faces in each version [Version 1: (5.33 ± 1.01) vs (2.47 ± 0.93), *t* (29) = 20.88, *p* < 0.001, Cohen’s *d* = 3.81; Version 2: (5.36 ± 1.01) vs (2.55 ± 0.94), *t* (29) = 19.67, *p* < 0.001, Cohen’s *d* = 3.59]. These two versions were well matched because no significant difference was observed in the smile intensity between the high-intensity smile faces across the two versions [(5.33 ± 1.01) vs (5.36 ± 1.01), *t* (29) = −0.66, *p* = 0.516, Cohen’s *d* = 0.12], nor between the low-intensity smile faces across the two versions [(2.47 ± 0.93) vs (2.55 ± 0.94), *t* (29) = −1.69, *p* = 0.102, Cohen’s *d* = 0.31].

### Trait Assessment Task Procedure

The participants were tested in a quiet and comfortable laboratory with good sound insulation. They were introduced to the trait assessment task and were informed that their task would be to rate a series of face photos using a list of trait adjectives. As previously described, two groups of face photos were used to create two equivalent versions of a program for rating traits. One of the two versions was randomly selected for each participant. Each version comprised eight trials in the practice stage and 726 trials in the formal experimental stage. The stimuli in the practice stage were selected from the Compound Facial Expressions of Emotion (CFEE) Database (Du et al., 2014). Eight Asian faces were randomly selected, including four neutral expressions and four happy expressions. The stimuli in the formal experimental stage were selected from the face photos of 66 people (focusing on expression intensity and gender information). The 726 trials comprised 11 blocks, in which the face photos of the 66 people were repeatedly presented 11 times. Each block was assigned so that the participants rated each of the 11 traits, namely trustworthiness, responsibility, attractiveness, sociability, confidence, intelligence, aggressiveness, dominance, competence, warmth, and tenacity on a 7-point Likert scale ranging from 1 (*not very ××*) to 7 (*very ××*).

Each block was presented in a random order among the participants. After completing each block, the participants rested for at least 60 s so that they could have a break before proceeding to the next experiment block. The entire experiment lasted for approximately 1 h.

## Results

The present study was designed as a 2 (expression intensity: high and low) × 2 (face gender: male and female) within-subjects design, and the dependent variable was the evaluation score of 11 traits: trustworthiness, responsibility, sociability, attractiveness, confidence, intelligence, aggressiveness, dominance, competence, warmth, and tenacity. The SPSS 24.0 (IBM, 2018) statistical software was used for data processing and analysis. First, Cronbach’s *α* coefficient was calculated to test the stability and consistency of the evaluations of different participants of each trait, which determined that all Cronbach’s alphas were above 0.77 (as shown in Table 3). This indicated that the evaluation scores of these 11 traits had good internal consistency, even though participants were judging different intensities of natural photographs (Nunnally, 1978).

**Table 3:** The Cronbach alphas of 11 trait rating scores.

Type of traits	High-intensity happy	Low-intensity happy
Trustworthiness	0.88	0.82
Responsibility	0.91	0.80
Sociability	0.88	0.77
Attractiveness	0.84	0.82
Confidence	0.87	0.85
Intelligence	0.88	0.82
Aggressiveness	0.93	0.88
Dominance	0.91	0.87
Competence	0.83	0.80
Warmth	0.93	0.87
Tenacity	0.86	0.87

Second, to verify the data satisfied the assumptions for the ANOVA, we had conducted a normal distribution test and homogeneity of variance test for the rating scores for each trait. The results indicated that the data satisfied normal distribution (Kolmogorov–Smirnov: *p* > 0.05) and Bartlett’s test of sphericity, which suggested that the ANOVA hypothesis had been satisfied. Therefore, the rating scores for each trait were analyzed separately in a 2 (happy expression intensity: high or low) × 2 (facial gender: male or female) repeated measures ANOVA. Because there were many dependent variables in this study, the probability of type I error through multiple comparisons might be increased. To decrease the probability of type I error, the significance thresholds for the *p*-values reported below were adjusted and the Bonferroni correction method of multiple tests was conducted according to the following formula: *α* = *α*/k (*α* = 0.05, *k* = 11). The difference was statistically significant with *p* < 0.0045 (as shown in Tables 4–6; Rezlescu et al., 2015).

**Table 4:** Evaluation scores for the different social traits under high- and low-intensity happy expressions (M ± SD).

Type of traits	Happy expression intensity		*F*	*p*	*η_p_*^2^
	High	Low			
Trustworthiness	4.54 ± 0.63	4.39 ± 0.51	1.28	0.2670	0.04
Responsibility	4.44 ± 0.74	4.83 ± 0.46	8.67	0.0060	0.22
Sociability	4.78 ± 0.64	4.06 ± 0.45	39.61	<0.0045	0.56
Attractiveness	3.70 ± 0.88	3.97 ± 0.64	3.97	0.0550	0.11
Confidence	4.83 ± 0.58	4.46 ± 0.55	8.52	0.0060	0.22
Intelligence	4.01 ± 0.66	4.45 ± 0.52	19.63	<0.0045	0.39
Aggressiveness	2.99 ± 0.82	3.69 ± 0.68	23.23	<0.0045	0.43
Dominance	3.40 ± 0.71	4.18 ± 0.68	21.53	<0.0045	0.41
Competence	4.21 ± 0.53	4.60 ± 0.47	14.76	<0.0045	0.32
Warmth	4.75 ± 0.80	4.05 ± 0.59	30.64	<0.0045	0.50
Tenacity	4.53 ± 0.61	4.71 ± 0.61	1.43	0.2410	0.04

*p < 0.0045 was considered statistically significant*.

**Table 5:** Evaluation scores for the different social traits under facial gender (M ± SD).

Type of traits	Facial gender		*F*	*p*	*η_p_*^2^
	Male	Female			
Trustworthiness	4.13 ± 0.55	4.80 ± 0.50	36.15	<0.0045	0.54
Responsibility	4.39 ± 0.53	4.88 ± 0.56	27.83	<0.0045	0.47
Sociability	4.48 ± 0.40	4.37 ± 0.60	1.50	0.2290	0.05
Attractiveness	3.62 ± 0.72	4.05 ± 0.70	20.91	<0.0045	0.40
Confidence	4.68 ± 0.43	4.61 ± 0.53	0.67	0.4180	0.02
Intelligence	4.18 ± 0.60	4.28 ± 0.55	1.22	0.2770	0.04
Aggressiveness	3.83 ± 0.79	2.85 ± 0.71	46.03	<0.0045	0.60
Dominance	4.09 ± 0.62	3.49 ± 0.53	36.33	<0.0045	0.54
Competence	4.26 ± 0.50	4.56 ± 0.46	12.04	<0.0045	0.28
Warmth	4.10 ± 0.69	4.70 ± 0.60	47.92	<0.0045	0.61
Tenacity	4.49 ± 0.52	4.75 ± 0.66	3.64	0.0660	0.11

*p < 0.0045 was considered statistically significant*.

**Table 6:** Evaluation scores for the different social traits under happy expression intensity and facial gender (M ± SD).

Type of traits	Happy expression intensity				*F*	*p*	*η_p_*^2^
	High		Low				
	Male	Female	Male	Female			
Trustworthiness	4.21 ± 0.78	4.88 ± 0.65	4. 50 ± 0.65	4.73 ± 0.61	0.03	0.8740	0.001
Responsibility	4.16 ± 0.75	4.72 ± 0.84	4.63 ± 0.55	5.03 ± 0.56	2.42	0.1300	0.072
Sociability	4.83 ± 0.59	4.74 ± 0.77	4.12 ± 0.49	4.00 ± 0.59	0.03	0.8600	0.001
Attractiveness	3.46 ± 0.92	3.94 ± 0.96	3.78 ± 0.73	4.16 ± 0.66	0.77	0.3870	0.024
Confidence	4.85 ± 0.65	4.82 ± 0.63	4.51 ± 0.52	4.41 ± 0.66	0.70	0.4080	0.022
Intelligence	3.93 ± 0.76	4.08 ± 0.66	4.43 ± 0.61	4.47 ± 0.60	1.03	0.3180	0.032
Aggressiveness	3.44 ± 1.04	2.55 ± 0.83	4.22 ± 0.78	3.16 ± 0.84	1.23	0.2770	0.038
Dominance	3.68 ± 0.89	3.12 ± 0.67	4.49 ± 0.80	3.86 ± 0.71	0.24	0.6300	0.008
Competence	4.01 ± 0.60	4.42 ± 0.56	4.50 ± 0.57	4.70 ± 0.58	4.07	0.0530	0.116
Warmth	4.45 ± 0.86	5.06 ± 0.82	3.76 ± 0.71	4.34 ± 0.61	0.07	0.7980	0.002
Tenacity	4.38 ± 0.70	4.69 ± 0.81	4.60 ± 0.66	4.81 ± 0.78	1.08	0.3080	0.034

*p < 0.0045 was considered statistically significant*.

For the judgments of trustworthiness, responsibility, and attractiveness, non-significant main effects of the happy expression intensity were observed, and the interaction between happy expression intensity and facial gender was not significant. However, the main effects of facial gender were significant, and the ratings of trustworthiness, responsibility, and attractiveness of the female faces were higher than those of male faces.

For sociability and intelligence, the main effects of the happy expression intensity were significant, and the ratings of sociability of the high-intensity happy faces were higher than the low-intensity happy faces, but the ratings of intelligence of the high-intensity happy faces were lower than the low-intensity happy faces. However, the main effects of facial gender and the interaction between happy expression intensity and facial gender were not significant.

For warmth, aggressiveness, dominance, and competence, the main effects of the happy expression intensity and facial gender were significant. For the happy expression intensity, the ratings of the warmth of the high-intensity happy faces were higher than the low-intensity happy faces, but the ratings of aggressiveness, dominance, and competence of the high-intensity happy faces were lower than the low-intensity happy faces. For the facial gender, the ratings of warmth and competence of the female faces were higher than the male faces, but the ratings of aggressiveness and dominance of the female faces were lower than the male faces. However, the interaction between happy expression intensity and facial gender was not significant.

For confidence and tenacity, the main effects of the happy expression intensity, facial gender, and the interaction between happy expression intensity and facial gender were not significant.

Further, this study reported the results of a one-sample *t*-test against the scale midpoints in addition to the relative comparisons between low- and high-intensity happy facial expressions (as shown in Tables 7 and 8).

**Table 7:** Evaluation scores for the traits under high-intensity happy expression and scale midpoints (M ± SD).

Type of traits	Facial expressions intensity		*T*	*p*	Cohen’s *d*
	High	The scale midpoints			
Trustworthiness	4.54 ± 0.63	4.00 ± 0.00	4.91	<0.0045	1.76
Responsibility	4.44 ± 0.74	4.00 ± 0.00	3.37	<0.0045	1.21
Sociability	4.78 ± 0.64	4.00 ± 0.00	6.69	<0.0045	2.40
Attractiveness	3.70 ± 0.88	4.00 ± 0.00	−1.95	0.0610	0.70
Confidence	4.83 ± 0.58	4.00 ± 0.00	8.15	<0.0045	2.93
Intelligence	4.01 ± 0.66	4.00 ± 0.00	0.05	0.9580	0.02
Aggressiveness	2.99 ± 0.82	4.00 ± 0.00	−6.96	<0.0045	2.50
Dominance	3.39 ± 0.71	4.00 ± 0.00	−4.78	<0.0045	1.72
Competence	4.21 ± 0.53	4.00 ± 0.00	2.29	0.0290	0.82
Warmth	4.75 ± 0.80	4.00 ± 0.00	5.34	<0.0045	1.92
Tenacity	4.53 ± 0.61	4.00 ± 0.00	4.94	<0.0045	1.77

*p < 0.0045 was considered statistically significant*.

**Table 8:** Evaluation scores for the traits under low-intensity happy expression and scale midpoints (M ± SD).

Type of traits	Facial expressions intensity		*T*	*p*	Cohen’s *d*
	Low	The scale midpoints			
Trustworthiness	4.39 ± 0.51	4.00 ± 0.00	4.26	<0.0045	1.53
Responsibility	4.83 ± 0.46	4.00 ± 0.00	10.32	<0.0045	3.70
Sociability	4.06 ± 0.45	4.00 ± 0.00	0.82	0.421	0.29
Attractiveness	3.97 ± 0.64	4.00 ± 0.00	−0.23	0.818	0.08
Confidence	4.46 ± 0.55	4.00 ± 0.00	4.71	<0.0045	1.69
Intelligence	4.45 ± 0.52	4.00 ± 0.00	4.89	<0.0045	1.76
Aggressiveness	3.69 ± 0.68	4.00 ± 0.00	−2.61	0.014	0.94
Dominance	4.18 ± 0.68	4.00 ± 0.00	1.48	0.150	0.53
Competence	4.60 ± 0.47	4.00 ± 0.00	7.18	<0.0045	2.58
Warmth	4.05 ± 0.59	4.00 ± 0.00	0.46	0.646	0.17
Tenacity	4.71 ± 0.61	4.00 ± 0.00	6.54	<0.0045	2.35

*p < 0.0045 was considered statistically significant*.

Finally, a normal distribution test and homogeneity of variance test were conducted for the rating scores of each trait in both versions. The results indicated that the data satisfied normal distribution (Kolmogorov–Smirnov: *p* > 0.05) and homogeneity of variance (Levene’s Statistic: *p* > 0.05). SPSS 24.0 (IBM, 2018) was used to perform independent sample *t*-tests on the rating scores of each trait in both versions. The results showed no significant differences in either Version 1 or Version 2 (as shown in Table 9), indicating that the evaluation scores of traits in both versions were generally homogeneous.

**Table 9:** Evaluation scores for the different social traits under Version 1 and Version 2 (M ± SD).

Type of traits	Versions		*t*	*p*	Cohen’s *d*
	1	2			
Trustworthiness	4.49 ± 0.44	4.44 ± 0.41	0.35	0.7320	0.12
Responsibility	4.61 ± 0.57	4.67 ± 0.40	−0.33	0.7430	0.26
Sociability	4.43 ± 0.45	4.41 ± 0.46	0.13	0.8970	0.04
Attractiveness	3.75 ± 0.67	3.92 ± 0.66	−0.73	0.4710	0.04
Confidence	4.72 ± 0.44	4.57 ± 0.43	1.04	0.3080	0.12
Intelligence	4.30 ± 0.53	4.16 ± 0.52	0.77	0.4500	0.21
Aggressiveness	3.28 ± 0.68	3.41 ± 0.59	−0.57	0.5720	0.20
Dominance	3.73 ± 0.61	3.84 ± 0.39	−0.66	0.5120	0.17
Competence	4.43 ± 0.49	4.39 ± 0.34	0.24	0.8150	0.27
Warmth	4.45 ± 0.70	4.35 ± 0.49	0.44	0.6630	0.09
Tenacity	4.63 ± 0.54	4.61 ± 0.36	0.18	0.8620	0.34

*p < 0.0045 was considered statistically significant*.

## Discussion

Based on the trait assessment task, this study manipulated the intensity of happy expressions (high, low) of target faces to explore the effect of happy emotional intensity on the social perception of Chinese faces. The results indicated that compared to the low-intensity happy expression, the high-intensity happy expression led to an enhanced perceptual outcome of the traits related to approachability, such as sociability and warmth, but not trustworthiness. Furthermore, compared to the low-intensity happy expression, the high-intensity happy expression reduced the perceptual outcome of traits related to capability.

### The Effect of the Intensities of Happy Expressions on Approachability

The “approachability” dimension represents a welcoming behavioral tendency of the target face. Happy expressions not only indicate positive emotional states but also convey friendly behavioral tendencies (Montepare and Dobish, 2003). Researchers have suggested that the intensity of expression corresponds to the intensity of behavioral tendencies (Ekman et al., 1980). Similar to the “morality differentiation hypothesis” (Goodwin et al., 2014; Landy et al., 2016), the “approachability” dimension of Chinese faces also includes two sub-dimensions: warmth and trustworthiness.

The results of this study showed that the sociability and warmth of high-intensity happy faces were rated higher than low-intensity happy faces, supporting the results of previous studies (Harker and Keltner, 2001; Mehu et al., 2008). Toothy smiles convey the behavioral tendency of an expressive person to build social ties and higher social intentions (Mehu et al., 2008; Bell et al., 2017) as well as increase the sense of friendliness, approachability, and warmth of the individual. Thus, it is believed that positive traits associated with social skills (e.g., sociability and warmth) tend to increase with the intensity of happy expressions. Some researchers believe that the positive effects of happy expressions of different intensities on the social perception of faces are derived from the baby-face overgeneralization effect, indicating that people tend to believe that adults with baby-face facial features have the same traits as infants, such as meekness, innocence, and enthusiasm. The intensity of happy expressions is associated with zygomatic muscle intensity (Wang et al., 2015). The typical facial features of high-intensity happy expressions (i.e., a widened nose, upturned mouth, shortened chin, and round face) are similar to the face of a baby (e.g., small, round, and small jaw; Dou et al., 2014). With the increase in the intensity of a happy expression, the facial features become more similar to the face of a baby (Walker et al., 2011; Wang et al., 2015), and the baby-face overgeneralization effect is more obvious. Therefore, the score of high-intensity happy expressions is higher than that of low-intensity happy expressions for sociability and warmth.

In addition to the “warmth” dimension, the “approachability” dimension of Chinese faces also includes the subdimension of trustworthiness, which is a representative trait of valence and includes responsibility, attractiveness, and confidence (Oosterhof and Todorov, 2008). The intensity of a happy expression did not affect the rated scores for trustworthiness, attractiveness, confidence, or responsibility. Based on the perceptual fluency hypothesis (Westerman et al., 2015), happy expressions of different intensities (positive emotional valence) correspond with the valence of trustworthiness, responsibility, attractiveness, and confidence (positive traits). The perceptual process is simple and does not vary with the intensity of happy expressions. However, the result for trustworthiness was inconsistent with previous studies, which suggested that children could perceive different levels of face trustworthiness based on cues of happy expressions of different intensities (25 and 50%), and the influence of happy expressions on trustworthiness perception would be enhanced with an increase in emotional intensity (Hess et al., 2000; Caulfield et al., 2014, 2016). There may be several reasons for this conflicting result. First, the experimental materials used in previous research comprised a combination of neutral and happy facial images (Hess et al., 2000; Caulfield et al., 2016); this could have caused the happy faces to appear less natural and the less intense happy face to appear even less natural, thus decreasing its trustworthiness rating. Second, the differences in interpretations of trustworthiness, compared with previous studies, might have explained the inconsistencies of the abovementioned study. Some researchers believed that the meanings of trustworthiness, warmth, and sociability are similar and that they are used to evaluate the friendly behavior intentions of the target face and that they are associated with communality (Hess et al., 2000; Fiske et al., 2007; Oosterhof and Todorov, 2008; Caulfield et al., 2014, 2016). However, in Chinese culture, trustworthiness refers to a moral norm that is associated with correctness rather than with the development of interpersonal skills (Shu et al., 2017), which supports the “morality differentiation hypothesis” (Goodwin et al., 2014; Landy et al., 2016). A highly sociable individual may not be perceived as being more trustworthy.

This result of this study regarding the effect on attractiveness was also inconsistent with the results of previous studies, which reported a positive correlation between the intensity of natural smiles and ratings on physical attractiveness (Golle et al., 2014). The main reason for this inconsistency might be due to the technique of stimulus creation. According to the “average hypothesis,” the degree of facial averageness is the main factor affecting facial attractiveness, and the more average the face, the higher the facial attractiveness (Li and Cheng, 2010). Previous studies have used average faces formed by the Psychomorph software instead of natural faces to explore the effect of smiling intensity on attractiveness. Such a design allowed the influence of both averageness and smiling intensity, thus making it impossible to distinguish the effect of facial averageness and smiling intensity on facial attractiveness (Golle et al., 2014). When facial averageness in the present study was controlled, the smiling intensity did not influence facial attractiveness.

The non-significant effect on the trait of responsibility might be due to its uniqueness. Responsibility refers to a positive trait characterized by effort, self-discipline, carefulness, and conscientiousness (Oosterhof and Todorov, 2008; Huang et al., 2014). Thus, its rating scores mainly reflect the executive power of the behavior rather than the behavioral tendency of the target individual, which might be less related to smile.

### The Effect of the Intensities of Happy Expressions on Capability

The result of the facial evaluation of people pertaining to the “capability” of a person represents the judgment of the ability of behavior intention of the target faces. Wu et al. (2020) found that the “capability” dimension denoted the traits of dominance and tenacity, which included physical and intellectual strength.

The results of this study showed that low-intensity smiling faces were rated as more dominant, aggressive, competent, and intelligent than high-intensity smiling faces. In general, the scores of physical strength, including dominance and aggressiveness, and intellectual strength, including competence and intelligence decrease with the increase in the intensity of happy expression. This is because the “capability” dimension is usually related to the attainment of military/political status, and the score of this dimension reflects the competitiveness and control of an individual in a particular field (Cheng et al., 2013). Compared to low-intensity smiling faces, high-intensity smiling faces have more baby-face features, and these faces represent weaker control (Kraus and Chen, 2013) and weaker competitiveness and competence (Gao et al., 2016). Additionally, regarding cultural differences, compared with Western leaders, Chinese leaders always present a calm and weak smile (Tsai et al., 2016), with more emphasis on “smiling without showing teeth” (Fang et al., 2019). In China, the expression of smiling without showing teeth is more likely to be a facial cue of high competence and dominance traits.

However, even if the overall trend is the same, due to the different meanings between dominance and competence, the intensity of happy expressions is not consistently evaluated for the two traits. This could be because dominance and aggressiveness are traits representing physical strength and imply a threatening ability to carry out the intention to hurt others, thus sharing a negative correlation with valence. Therefore, the dominance and aggressiveness scores for happy faces are lower than or equal to the scale midpoints. Compared to low-intensity happy faces, high-intensity happy faces increase the propensity for submissive behaviors. When people desire to build cooperative relationships with others (Mehu et al., 2008; Bell et al., 2017), or are in search of rapport (Hennig-Thurau et al., 2006), they tend to smile more intensely. This submissive motivation is also incompatible with the characteristics of the dominance trait (threat). Therefore, the scores of dominance and aggressiveness decrease with the increase in the intensity of happy expression. On the other hand, people with high competence gain social status through a high level of ability or generosity, and there is a positive correlation between competence and valence. Therefore, competence including intelligence scores for happy faces is greater than or equal to the median. However, high-intensity smiling faces are often considered to show that people are carefree, satisfied with the status quo, and open to change and improvement (Bodenhausen et al., 1994). This is inconsistent with the intention conveyed by the component of competence (e.g., high creativity and high efficiency; Fiske et al., 2007); thus, high-intensity happy expressions might be facial cues for a lack of competence. In addition, target faces were found to be affected by a stronger positivity effect in the competence domain for moderate levels of behaviors (Rusconi et al., 2020). Therefore, compared to high-intensity happy expressions, low-intensity happy expressions that are attributed to moderate levels of behaviors might work as to be facial cues for competence.

However, the present study demonstrated that the intensity of the happy expression did not affect the evaluation score of tenacity, though it was usually comprehended in the dimension of “capability.” As tenacity refers to a trait that is exhibited to protect the body from harm under stress (Zhang and Wang, 2011), a person with strong tenacity is more inclined toward focusing on problem-coping strategies than on emotion-coping strategies (Nicholls et al., 2008). Therefore, the score of tenacity might be unrelated to the intensity of happy expressions.

Taken together, the present studies have made a worthwhile contribution to the existing literature. In terms of the current research, this study explored how different intensities of happy expressions influenced the social perception of faces in the Chinese context. The results supported the “morality differentiation hypothesis” that trustworthiness and warmth/sociability had different meanings in China. Sociability in the context of Chinese culture focuses on the development of interpersonal skills (e.g., emotional management skills and conflict resolution strategies). Further, sociability is associated with communality (Zhang et al., 2012). However, trustworthiness is considered a moral code, and it is uniquely associated with correctness (Shu et al., 2017). Therefore, the intensity of happy expressions has different effects on these two traits. Compared with low-intensity happy expressions, high-intensity happy expressions only improve the evaluation score of sociability and do not affect the evaluation score of trustworthiness. Second, previous researchers have studied the “morality differentiation hypothesis,” which was applicable to the top-down stereotype content processing and familiar groups processing (Goodwin et al., 2014; Landy et al., 2016), as well as highlighted the distinct role of trustworthiness in face perception from the bottom-up perspective (Krumhuber et al., 2007; Todorov et al., 2015; Todorov and Oh, 2021). Compared with the previous studies, the present study distinguished trustworthiness and sociability through trait assessment tasks in the first impressions of strangers with different intensity smiling, which added another supportive evidence for the “morality differentiation hypothesis.” Third, the present study used natural face photographs, thus having more ecological validity than computer-generated faces and composite images that were used in previous studies, revealing the novel finding of this study that the trustworthiness and attractiveness ratings were not affected by the intensity of happiness. Fourth, the present study showed the differences between physical and intellectual strength. For example, the physical strength rating for low-intensity happy expressions was equal to the scale midpoints, while the score of intellectual strength was higher than the scale midpoints; similarly, for high-intensity happy expression, the physical strength rating was lower than the middle value, and that for intellectual strength had no significant difference from the scale midpoints. Fifth, the present study fully described how the influence of the intensity of happy expressions influenced 11 traits: trustworthiness, responsibility, attractiveness, sociability, confidence, intelligence, aggressiveness, dominance, competence, warmth, and tenacity. This has consequently provided more practical suggestions for the daily communications of people, as well as hints for researchers who are interested in conducting further research on one or several traits.

Although the present study produced several interesting findings, it has several limitations. First, this study only selected happy expressions, thereby lacking negative and neutral expressions for comparison groups. Further research must compare the effects of positive, negative, and neutral expressions on personality trait assessment. Second, this study adopted a within-subjects design that is similar to the studies of Walker et al. (2011) and Wang et al. (2019), in which the bias of the perceiver on the social perception of faces can be controlled; however, the evaluation of one trait by the participants was found to affect their judgment of another trait. To control the judgment error of traits by the same participants, the 11 traits in this study were divided into 11 blocks and then presented in random order to the participants. A mandatory rest time of 60 s was also set between each block for participants, as well as a freely regulated rest time. Therefore, the influence of the evaluation of the same participant of one trait that could affect the judgment of another trait was controlled, and the fatigue of the participants was also reduced. Future studies should adopt a between-subjects design to verify the stability of the results of this study. Third, because there are many levels of dependent variables in this study, multiple statistical analyses were conducted. Although they were statistically corrected, this does not eliminate the possible misrepresentation or understatement effect caused by multiple statistical comparative analyses. Future studies should conduct further targeted tests on these effects. Fourth, this study addressed the gap in previous research by considering how different intensities (low vs. high) of happy facial expressions affected the ascription of 11 traits focusing on Chinese faces. However, this current study lacked a direct comparison between Chinese and Western faces and participants. Therefore, further research that directly compares the underlying cultural differences of how different intensities of happy expressions affect the social perception of faces is necessary. Fifth, this was an exploratory experiment, and future research needs to recruit more participants to replicate the results of this study.

## Conclusion

In summary, the present study revealed that different intensity happy expressions (high-intensity or low-intensity) had different effects on the social perception of Chinese faces among Chinese participants. This was mainly manifested by high-intensity happy expressions receiving higher scores for sociability and warmth in the dimension of “approachability,” as compared with low-intensity happy expressions. Further, high-intensity happy expressions had lower scores for the dimension of “capability,” (e.g., dominance, competence, and intelligence).

## Data Availability Statement

The original contributions presented in the study are included in the article/supplementary material, further inquiries can be directed to the corresponding authors.

## Ethics Statement

The studies involving human participants were reviewed and approved by the Ethics Committee of Liaoning Normal University. The patients/participants provided their written informed consent to participate in this study. Written informed consent was obtained from the individual(s) for the publication of any potentially identifiable images or data included in this article.

## Author Contributions

ZJ conceived this study. YL and YY participated in writing and revising the manuscript. YL and HL participated in performing the study. FP and QW participated in modifying the manuscript. All authors contributed to the article and approved the submitted version.

### Conflict of Interest

The authors declare that the research was conducted in the absence of any commercial or financial relationships that could be construed as a potential conflict of interest.
